# Restoring Mitochondrial Function: A Small Molecule-mediated Approach to Enhance Glucose Stimulated Insulin Secretion in Cholesterol Accumulated Pancreatic beta cells

**DOI:** 10.1038/srep27513

**Published:** 2016-06-10

**Authors:** Suman Asalla, Shravan Babu Girada, Ramya S. Kuna, Debabrata Chowdhury, Bhaskar Kandagatla, Srinivas Oruganti, Utpal Bhadra, Manika Pal Bhadra, Shasi Vardhan Kalivendi, Swetha Pavani Rao, Anupama Row, A Ibrahim, Partha Pratim Ghosh, Prasenjit Mitra

**Affiliations:** 1 Dr. Reddy’s Institute of Life Sciences, University of Hyderabad Campus, Gachibowli, Hyderabad, Telengana, 500046, India; 2 Dept. of Biochemistry, School of Life Sciences, University of Hyderabad, Gachibowli, Hyderabad, Telangana, 500046, India; 3 Indian Institute of Chemical Technology, Uppal Road, Tarnaka, Telengana, Hyderabad, 500007, India; 4 Center of Cellular and Molecular Biology, Habsiguda, Uppal Road, Hyderabad, 500007, India; 5 University of Hyderabad Health Center, University of Hyderabad, Gachibowli, Hyderabad, Telengana, 500046, India; 6 Department of Biochemistry, National Institute of Nutrition, Hyderabad 500007, India; 7 Microsoft India (R&D) Pvt. Ltd, Gachibowli, Hyderabad, Telengana, 500032, India

## Abstract

Dyslipidemia, particularly the elevated serum cholesterol levels, aggravate the pathophysiology of type 2 diabetes. In the present study we explored the relationship between fasting blood sugar and serum lipid parameters in human volunteers which revealed a significant linear effect of serum cholesterol on fasting blood glucose. Short term feeding of cholesterol enriched diet to rodent model resulted in elevated serum cholesterol levels, cholesterol accumulation in pancreatic islets and hyperinsulinemia with modest increase in plasma glucose level. To explore the mechanism, we treated cultured BRIN-BD11 pancreatic beta cells with soluble cholesterol. Our data shows that cholesterol treatment of cultured pancreatic beta cells enhances total cellular cholesterol. While one hour cholesterol exposure enhances insulin exocytosis, overnight cholesterol accumulation in cultured pancreatic beta cells affects cellular respiration, and inhibits Glucose stimulated insulin secretion. We further report that (E)-4-Chloro-2-(1-(2-(2,4,6-trichlorophenyl) hydrazono) ethyl) phenol (small molecule M1) prevents the cholesterol mediated blunting of cellular respiration and potentiates Glucose stimulated insulin secretion which was abolished in pancreatic beta cells on cholesterol accumulation.

Type 2 diabetes accounts for the majority of the cases of diabetes and is characterized by insulin resistance and pancreatic beta cell dysfunction^1^ resulting in the alteration of glucose homeostasis. Although insulin resistance and systemic inflammation contribute to the patho-physiology of the disease^2^,^3^,^4^, pancreatic beta-cell dysfunction and consequent impaired Glucose Stimulated Insulin Secretion (GSIS) is considered to be an essential step for the progression of the disease from pre-diabetic to the diabetic stage^1^,^5^. The resultant hyperglycemia is influenced by several co-morbidities like hyperlipidaemia^6^,^7^, hypercholesterolaemia^8^,^9^, and elevated plasma triglycerides^10^ which contribute to the metabolic signaling that regulate GSIS^11^.

Recent evidences propose the role of cholesterol homeostasis in maintaining the adequate secretory response of insulin from pancreatic beta cells^12^. Mice with pancreatic beta cell specific knock out of ATP-binding cassette transporter subfamily A member 1 (ABCA1), the regulator of cholesterol efflux, shows impaired GSIS^13^. Patients suffering from Tangier disease, caused by the deficiency of ABCA1, have attenuated GSIS reflecting on the importance of cholesterol efflux from pancreatic beta cells for the maintenance of proper insulin secretory response^14^. Further, the tissue specific knock out of ATP-binding cassette transporter G1 (ABCG1), the protein which alters the intracellular cholesterol distribution, has been shown to impair GSIS in pancreatic beta cells^15^. Hao *et al*. had developed genetically engineered mouse model where increase in the serum cholesterol without any alteration of serum free fatty acids has been shown to attenuate GSIS^8^. These data indicate the importance of proper intracellular cholesterol distribution in regulating pancreatic beta-cell function. In the present study, we carried out a cross sectional analysis of human samples using Support Vector Regression (SVR) method^16^ to assess the influence of serum cholesterol on fasting blood sugar in normal healthy volunteers and type 2 diabetes patients. The result of the present study documented that low density lipoprotein (LDL) and serum cholesterol have a **linear** impact on fasting hyperglycemia indicating their influence on the progression of the disease from pre-diabetic to the diabetic state. The impact of serum cholesterol on insulin exocytosis was further assessed in diet induced hypercholesterolemic animal model, where rodents on short term feeding with hypercholesterolemic diet showed hypercholesterolaemia and hyperinsulinemia with modest elevation of blood glucose. To explore how cholesterol enrichment modulates the GSIS, we developed a cell based model by treating BRIN-BD11 pancreatic beta cells with soluble cholesterol. The data presented in this study shows that cholesterol exposure causes total cholesterol accumulation, blunts cellular respiration and compromises GSIS. We also provide evidences that pre-treatment with the small molecule M1 stimulates cellular respiration, restores the mitochondrial function and stimulates GSIS which was abolished in cholesterol treated pancreatic beta cells.

## Results

### Total cholesterol and its impact on fasting blood sugar in human subjects

We analyzed the approximate relationship (‘*f*’ as below) in human population involving the following multi-dimensional regression problem (equation 1)


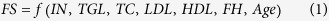


Here FS stands for fasting blood sugar; IN: insulin; TGL: Triglycerides; TC: Total cholesterol, LDL: Low Density Lipoproteins; HDL: High density Lipoproteins and FH: Family History.

Typically, a well approximated function is the one that minimizes the root mean square error (RMSE) over the set of candidate functions. In the present case we employed a variant of Support Vector Regression (SVR) model (ν-SVR) by fitting a set of training data, *T*, of dimension ‘d’, to the model as shown in equation (2).

Thus,


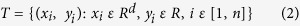


For the problem in hand, ‘*R*’ is the real line, *d* = 7, *n* = 144 (supplemental Table 1), ‘*x*_*i*_’ is the *i*^*th*^ sample from a human volunteer represented as a tuple in 7-dimensional input space. ‘*y*_*i*_’ represents the *i*^*th*^ value of FS given ‘*x*_*i*_*’ i.e.* y_*i*_ = f (x_i_) as in (1). Support Vector Machines developed by Cortes and Vapnik *et al*.^17^ has recently been employed extensively in biological prediction problem^18^, clinical decision making^19^ & risk prediction of common diseases like prediabetes and diabetes^20^. To provide a background of the underlying theory, such problems are typically modelled as quadratic optimization problems. The algorithm tries to fit an optimal hyper plane separating multiple classes of training data points by identifying support vectors. The risk functional (ψ) associated with such problems is defined as follows (equation 3):


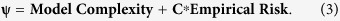


For a hyperplane, the model complexity is proportional to ′***w***^***T***^***w***′ where ‘***w**’* is the normal vector to the hyperplane or coefficient vector. Additionally, a quantity called *Empirical Risk, R*_*emp*_ *(f)*, is defined which measures the classification error of the training samples. ‘*C*’ is the penalty factor for, *R*_*emp*_ *(f)*. Hence, for a SVM class of problems, ′*ψ*′ can be rewritten as:


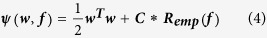


In equation (4), ‘*f*’ is the prediction function to be estimated and ‘*C*’ is typically pre-specified and/or experimented for optimal solution.

Using one of the common forms of *R*_*emp*_(*f*) [4], we can state the general SVM classification problem as follows:


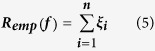



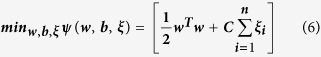


The above objective function is subjected to the following constraints:


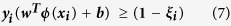



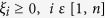


*ξ*_*i*_: Misclassification error of the *i*^*th*^ training sample.

*b* *ε* *R*: Intercept of the separating hyper plane.

Equation 7 has been reformulated in the above to deal with the nonlinearity of the input space by introducing a map, ‘ϕ’. It helps flatten the non-linear input space by enhancing the dimensions of the input space in a principled manner **(Kernel trick)**. As a consequence, the training vectors (x_i_) in the resultant space gets separated out in the resultant higher dimensional space. This enables training vectors to be classified using linear classifiers in majority of the situations.

Equation (6) actually represents a quadratic minimization problem in its primal form. Typically, this is reduced to its dual form as follows (equation 8):


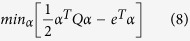


The equation (8) is subject to the following constraints:


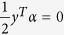


where,


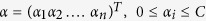



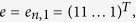



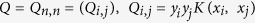



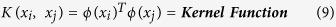


In our present analysis, the risk functional for a general SVR regression problem [3] is defined as follows:


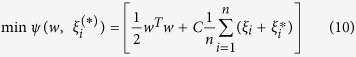


In the above, 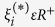 represents both (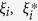) in short form and is called the estimation error.


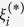
s are actually slack variables which enhance the chances of obtaining feasible solutions and are similar to the ‘soft margin’ concept in SVM classification. We can extract *R*_*emp*_(*f*) from **(10)** as follows:


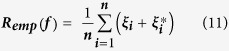


The introduction of 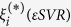 in the above effectively implements an ′*ε*′ insensitive loss function. Here the penalty incurred is zero as long as the predicted value remains within the ′*ε*′ limit of the target value thus discouraging large errors.

We also applied nu-SVR (‘ν-SVR’) technique to determine the risk functional modifying equation 11 as follows:


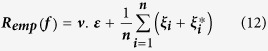


The primal problem, hence, can be stated as follows:


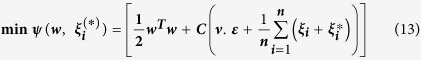


The above objective function is subjected to the following constraints using nonlinear mapping ‘*ϕ*’:


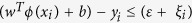



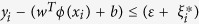



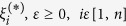



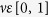


The range of values ′*ν*′ can take is (upper) bounded by the fraction of margin errors and the (lower) bounded by the fraction of support vectors. Asymptotically both the bounds converge *“almost surely”* to one.

**Figure 1:**
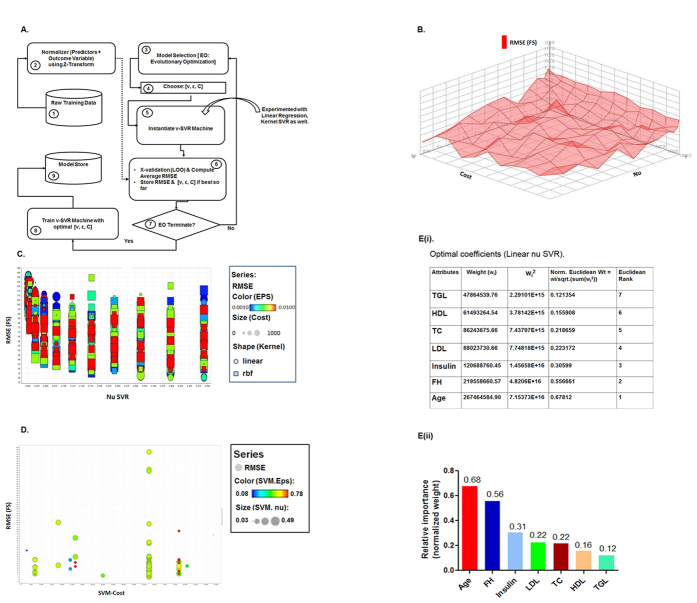
Effect of total cholesterol and LDL on Fasting Blood Sugar in surveyed Indian population. (**A)** Architecture of parameter optimization for the support vector regression machines. The number represents the steps in the flow diagram. Dotted arrow represents the normalized data which is used by cross validation process along with instantiated SVR machine. Straight arrow designates the sequence flow of the experiment. (**B)** Plot of RMSE for a linear nu-SVR with other parameters remaining constant, X-Axis: [values of nu], Y-Axis: Cost (C), Z-Axis: RMSE (FS). (**C)** Plot of RMSE (FS) described as a function of nu SVR with other parameters [Kernel Type, Epsilon, Cost]. X-Axis: Nu, Y-Axis: RMSE (FS), Color Code: Epsilon, Size (0–100): Cost (C), Shape: Kernel function; circular: Linear Kernel; square: Radial Basis Kernel (RBF). (**D**) Plot of RMSE (FS) generated through LOO cross validation using Evolutionary Computational Approach search technique. X axis: SVM-Cost (C); Y-Axis: RMSE (FS), Color Code: Epsilon, Size: Nu. (**E)** Optimal coefficients of linear nu SVR describing the slope of the optimal hyperplane against each variable (weights) ; normalized relative weights of the predictors including the rank as expressed in the charts (**E(i))** as well as histogram **((E(ii))** depicting relative impact of predictors on fasting blood sugar (FS).

In this present study, the quest for optimal estimation of the function ‘f’, has been achieved by navigating through the parameter space [Cost (C): 0–1000, Epsilon: 0.0001–1, nu: 0.001–0.5, Gamma: 0–1, Kernel Types: {‘linear’, ‘rbf’}, Step Size = 10] across 29,242 SVR configurations by performing brute force grid search. Figure 1A describes the architecture of the regression machine constructed by support vector algorithm. The linearity of the outcome variable, FS was established as a function of the remaining seven predictors as we looked for the optimal RMSE (Root Mean Square Error) value. The RMSE plot of the FS over the entire configuration space has a very rough terrain with plenty of local optimum as shown in Fig. 1B. The RMSE value has been plotted in Fig. 1C as a function of ′*ν*′ where linear kernels are the ones with circular shapes while rectangular shapes reflects the rbf (radial basis function) kernels. The value of the epsilon parameter that resulted in the optimal predictor performance was within the middle of the range [0.001, 0.01] with a cost parameter value of (C = 800). This also confirms that the characteristics exhibited by a predictor function in equation 1 are essentially linear as a function of seven biological parameters.

To ensure robustness further, we performed grid search by expanding search over the parameter space by varying [Cost (C): 0–100000, Epsilon: 0.0001–1, nu: 0–0.5] using Evolutionary Computation approach^21^ and introducing Leave one out cross validation (LOOCV) technique for RMSE estimation. This is a much improved process over the brute force grid search method as the algorithm converges much faster and typically with much better local optimum i.e. RMSE in our case. Several conclusions were evident as shown in Fig. 1D,E. First and foremost, linear relationship prevailed among the predictors and outcome variables as we expanded the parameter search space. Secondly, the order of normalized weights of the predictors remained exactly the same as in the case of brute force grid search. Finally, we got better RMSE (36.43) as we expanded the search.

For completeness, we also experimented using simple linear regression technique (RMSE: 37.063) as well as RBF (RMSE: 42.190) non-linear techniques just to establish that the linear nu-SVR approach outperforms all the methods (linear, nonlinear) considered so far.

A detailed analysis of the results thus indicated that the relationship (Eq. 1) exhibited by the data set is essentially linear. For the given data set, optimal prediction is provided by a linear nu-SVR model with parameters *C* = 62338.06, *ν* = 0.44 *and ε* = 0.583 *and an RMSE* = 36.430. The RMSE plot for the experiment over the entire configuration space was summarized in Fig. 1D where the RMSE value has been plotted as a function of ′C′for the linear SVR; ′*ε*′ has been color coded and size of the data points are modulated by ′*ν*′.

The importance (*w*_*i*_) of each predictor reflects the degree to which they influence prediction of FS. These weights have been normalized such that,


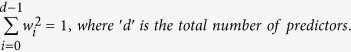


Each of the *w*_*i*_ s actually represents the tangent of the angle subtended by the predictor with the outcome variable (FS). The *w*_*i*_ also captures the amount of change in FS outcome variable for a unit change in a predictor keeping the remaining predictor values unchanged. The normalized weights are arranged in order as represented in a chart (Fig. 1E(i)) and histogram (Fig. 1E**(ii)),** which clearly reveals that fasting blood sugar (FS) is significantly influenced by ‘Age’, followed by ‘FH’, ‘IN’, ‘LDL’, ‘TC’, ‘HDL’ and ‘TGL’.

### Rodent model on hypercholesterolemic diet shows elevated serum cholesterol and serum insulin

In the next step we investigated whether animal models with elevated serum cholesterol have any impact on fasting blood sugar. We designed experimental diet for Sprague Dawley rats comprising of 20% Peanut oil, 1% cholesterol, standard nutrients with fructose as a source of carbohydrate (hypercholesterolemic diet, experimental diet 3 in Table 1) and compared with the group having experimental diets containing 20% peanut oil and standard nutrients with starch or fructose as a source of carbohydrate (experimental diet 1and 2, Table 1). Peanut oil was chosen as a source of fat as the majority of the human population assessed in this study was found to use peanut-oil cooked food as staple diet. Our data reveals that the Sprague Dawley rats when fed on hypercholesterolemic diet for 3 months exhibit significant accumulation of total cholesterol (from 1.16 ± 0.07 to 1.65 ± 0.14 mg/gram tissue) in islets, elevated serum insulin and serum cholesterol with a modest increase in blood glucose level (Table 2). The data is in concert with the model in human subjects which showed correlation between elevated serum cholesterol and enhanced fasting blood sugar.

### Cholesterol exposure on pancreatic beta cell: impact on Glucagon-like peptide 1 Receptor- mediated cAMP generation

**Figure 2:**
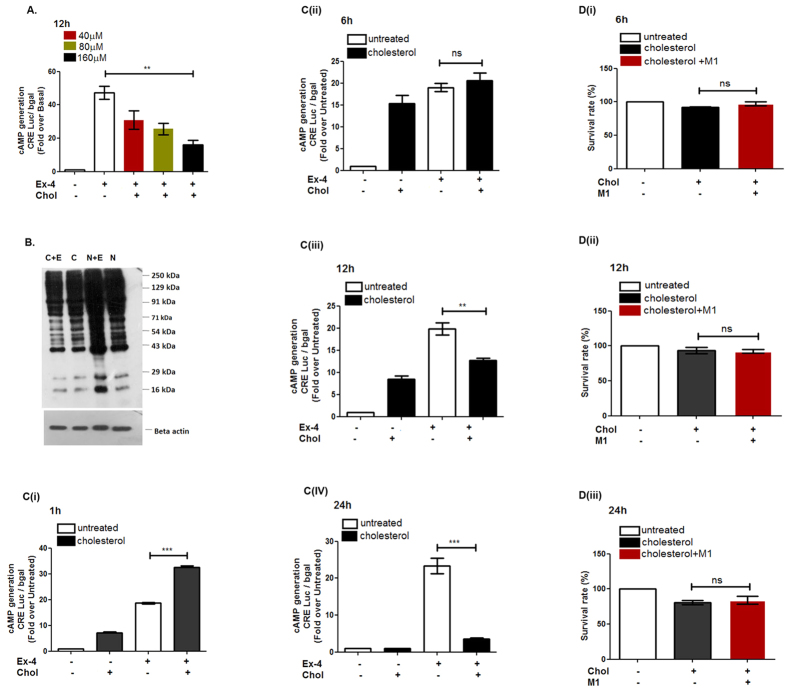
Effect of soluble cholesterol on GLP-1R mediated cAMP generation. (**A)** GLP-1R mediated cAMP generation in pancreatic beta cells treated with different concentration of cholesterol for 12 h and cAMP generated was measured by luciferase assay(CRE: cyclic AMP responsive element). (**B)** PKA substrate phosphorylation profile in normal and cholesterol treated pancreatic beta cells; C: cholesterol treated, C + E: cholesterol and Exendin-4 treated, N: Untreated; N + E: Exendin-4 treated. (**C)** Time course analysis of the effect of soluble cholesterol on basal and GLP-1R mediated cAMP generation. The cells were treated with 160 μM cholesterol for 1 h (**C(i)**), 6 h (**C(ii)**), 12 h (**C(iii)**) and 24 h (**C(iv)**) following which treated with GLP-1R agonist and cAMP generated was measured by luciferase assay. (**D)** Time course analysis of the effect of soluble cholesterol on survival of BRIN-BD11 pancreatic beta cells: the cells were treated with 160 μM soluble cholesterol for 6 h (**D(i)**), 12 h (**D(ii)**) and 24 h (**D(iii)**), following which the cell survival was measured by MTT assay. Data are shown as mean ± SEM, n ≥ 3; *P < 0.05, **P < 0.01, ***P < 0.001 *t*-test (unpaired), ns: not significant; Ex-4: Exendin-4; chol: cholesterol; M1: small molecule M1.

Cholesterol accumulation in pancreatic islets and the resultant hyperinsulinemia led us to evaluate the mechanism by which cholesterol enrichment in pancreatic beta cells modulate insulin exocytosis. Intracellular cholesterol accumulation modulates the membrane micro-domains that are the critical hub for initiation of signaling events^22^. Since Glucagon-like peptide 1 Receptor (GLP-1R) mediated generation of cAMP triggers a critical signaling cascade that regulates glucose stimulated insulin exocytosis, we explored how cholesterol modulates the GLP-1R stimulated cAMP generation in pancreatic beta cells. Cultured pancreatic beta cells were treated with 40 μM, 80 μM and 160 μM cholesterol for 12 h and evaluated for the modulation of cAMP generation. As Fig. 2A reveals, treatment of BRIN-BD11 pancreatic beta cells with 160 μM cholesterol for 12 h significantly decreases GLP-1R mediated cAMP generation, the effect being reflected in the reduction in the intensity of phosphorylation of PKA substrates which is a downstream signaling event corresponding to GLP-1R mediated cAMP generation (Fig. 2B).We next carried out a time course dependent evaluation of basal and GLP-1R mediated cAMP generation in pancreatic beta cells. The BRIN-BD11 pancreatic beta cells were treated with soluble cholesterol for 1 h, 6 h, 12 h and 24 h following which basal and GLP-1R mediated cAMP generation was assessed. As Fig. 2C(i) reveals, 1 h cholesterol treatment enhances basal cAMP generation by 7.14 ± 0.89 fold and GLP-1R mediated cAMP generation by 32.87 ± 0.89 fold with respect to untreated control whereas GLP-1R activation in cholesterol –untreated cells enhances cAMP generation by18.61 ± 0.61fold.We next evaluated the effect of 6 h cholesterol treatment on basal and GLP-1R mediated cAMP generation. The data shows that while basal cAMP generation is increased by 15 fold, there is no alteration of GLP-1R mediated cAMP generation in cholesterol treated and untreated BRIN-BD11 pancreatic beta cells (Fig. 2C **(ii)).** Figure 2C **(iii)** describes the effect of 12 h cholesterol treatment of cultured BRIN-BD11 pancreatic beta cells where basal cAMP generation was increased by 8.51 ± 1.49 fold but GLP-1R mediated cAMP generation was decreased from 19.82 ± 2.85 fold to 12.74 ± 0.93 fold. Treatment of BRIN-BD11 cells with 160 μM cholesterol for 24 h has no effect on basal cAMP generation (1.04 ± 0.04 fold w.r.t untreated control), however, GLP-1R mediated cAMP generation was drastically reduced from 23.32 ± 4.25 fold to 3.51 ± 0.85 fold (Fig. 2C**(iv)).** These data shows that basal and GLP-1R mediated cAMP generation is dependent on the period of cholesterol treatment; 12 h cholesterol treatment represents a specific time point where inspite of increase of basal cAMP generation, there is a significant decrease in GLP-1R mediated cAMP generation reflecting on the perturbation to GLP-1R signaling cascade due to cholesterol exposure. The generation of basal cAMP has been reported to inhibit apoptosis through cAMP Response Element Binding Protein (CREB)- mediated induction of insulin receptor substrate 2(IRS2)^23^ and through stimulation of inhibitor of apoptosis protein 2 (IAP-2)^24^. Our data is in concert with the published literature as we observed no cell death on treatment of pancreatic beta cells with 160 μM cholesterol for 6 h and 12 h (Fig. 2D(i,**ii)).** On the contrary, the 24 h time point, which showed no enhancement of cAMP generation due to the treatment with 160 μM cholesterol, revealed 19.04 ± 5.20% cell death as measured by the MTT assay. Thus, the lack of cell death and at the same time attenuation of GLP-1R mediated cAMP generation led us to consider 12 h time point suitable for further exploration to the mechanism of cholesterol mediated attenuation of GSIS.

### Cholesterol exposure for 12 h enhances total cholesterol and blunts GLP-1R mediated insulin exocytosis in pancreatic beta cells

**Figure 3:**
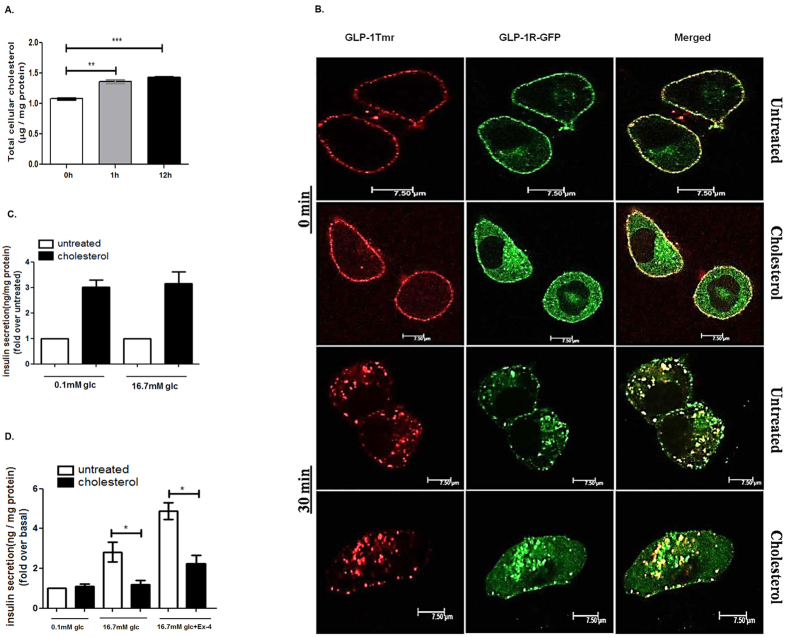
Cholesterol accumulation in pancreatic beta cells and its effect on GLP-1R mediated potentiation of GSIS. (**A)** The cells were incubated with 160 μM cholesterol for 1 h or 12 h following which total cholesterol is measured. Data are normalized as μg/mg protein and presented as mean ± SEM of 3 independent experiments. (**B)** Time-course analysis of GLP-1R internalization in control and cholesterol enriched BRIN-BD11 pancreatic beta cells. Cells were transfected with GFP tagged GLP-1R and stimulated with 1 μM GLP-1Tmr for 0 min and 30 min and visualized by confocal microscopy. Images are representative of three independent experiments.(Scale: 7.5 μm) (**C)** Insulin exocytosis from BRIN-BD11 pancreatic beta cells following 1 h treatment with soluble cholesterol in presence of 0.1 mM glucose and 16.7 mM glucose. Insulin secretion was measured as ng insulin /mg protein and the data was expressed as fold increase over untreated control (basal insulin secretion at 0.1 mM glucose as well as basal insulin secretion at 16.7 mM glucose is considered as 1 fold and the respective cholesterol mediated insulin exocytosis is compared). (**D)** GLP-1R mediated potentiation of GSIS in cholesterol enriched pancreatic beta cells. Control and cholesterol enriched BRIN-BD11 cells were treated with GLP-1R agonist Exendin-4(Ex-4) in presence of 16.7 mM glucose and insulin secretion was measured as ng insulin/mg protein for a period of 30 min. Insulin secretion from untreated cells in presence of 0.1 mM glucose is considered as basal. Data are expressed as fold increase of insulin secretion over basal secretion and presented as mean ± SEM of 4 independent experiments.

In the next step we analyzed whether exposure to soluble cholesterol resulted in the elevation of cellular cholesterol in pancreatic beta cells. Our data shows that both 1 h and 12 h soluble cholesterol exposure enhanced total cellular cholesterol concentration by 1.26 ± 0.05 (p < 0.05) and 1.33 ±0.04 fold (p < 0.001) (Fig. 3A). The intracellular cholesterol content was found to be enhanced by 1.268 ± 0.048 fold in subcellular fraction enriched with mitochondria, endosomes and secretory granules (supplementary Fig. 1a). A comparable increase of total cholesterol in pancreatic islets with the increase of serum cholesterol was observed in rats fed on hypercholesterolemic diet (Table 2) as was also reported by Hao *et al*. in their work with islets obtained from genetically engineered mouse models^8^.

Previous report from our laboratory described for the first time that prevention of GLP-1R internalization reduces cAMP generation and blunts the GSIS in pancreatic beta cells^25^. Since cholesterol accumulation alters the membrane microdomains, we explored whether increase in cellular total cholesterol modulates the ligand interaction and subsequent internalization of GLP-1R. As Fig. 3B reveals, increase in cellular total cholesterol content has no impact on internalization of GLP-1receptor- ligand complex indicating that GLP-1R trafficking on ligand binding remains uninterrupted on increase in total cellular cholesterol in pancreatic beta cells.

Bogan *et al*., reported the deposition of cholesterol in insulin granules which has been shown to impede insulin granule exocytosis in MIN6 insulinoma cells^26^. In our present study, we assessed whether 1 h or 12 h treatment with 160 μM soluble cholesterol affects insulin exocytosis in pancreatic beta cells. The cholesterol treatment for 1 hour significantly increases insulin exocytosis by 3.01 ± 0.46 fold over untreated control in the presence of 0.1 mM glucose and 3.15 ± 0.81 fold in the presence of 16.7 mM glucose (Fig. 3C). The increase is consistent with the enhancement of both basal and GLP-1R mediated cAMP generation on cholesterol exposure which may activate PKA and EPAC-2 signaling pathways; however, a 12 h exposure of cultured pancreatic beta cells to 160 μM soluble cholesterol reduces GSIS drastically from 2.82 ± 0.87 fold to 1.18 ± 0.40 fold (p < 0.05) and GLP-1R induced insulin secretion (GIIS) from 4.87 ± 0.73 fold to 2.23 ± 0.76 fold without compromising basal insulin secretion (Fig. 3D). The decrease of GSIS and GIIS is despite of a 12.74 ± 0.93 fold increase of cAMP generation over untreated basal on GLP-1R activation in cholesterol accumulated pancreatic beta cells. The data points at the involvement of additional cellular events that modulates GSIS in pancreatic beta cells during pathophysiological condition of cholesterol accumulation.

### Cholesterol accumulation in pancreatic beta cells impairs mitochondrial function

**Figure 4:**
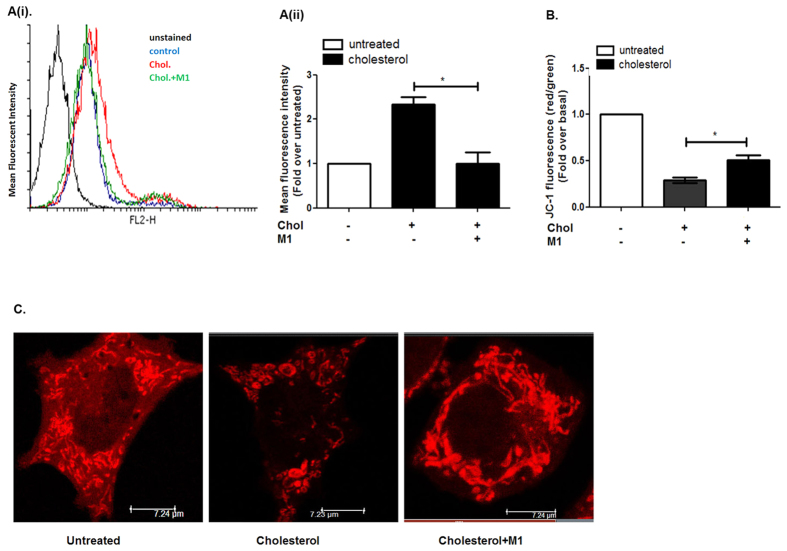
Cholesterol accumulation and its impact on mitochondrial function and architecture. (**A)** Measurement of mitochondrial ROS in control and cholesterol treated pancreatic beta cells with or without the pre-treatment of small molecule M1. The cells were stained with Mito SOX red for 30 minutes and analyzed by flow cytometry. (**A(i)**) Representative histogram showing mean fluorescence intensity of MitoSOX stained cells; **Black:** Untreated, Unstained, **Blue:** Untreated stained, **Red:** Cholesterol treated, **Green:** M1pre-treated cells treated with cholesterol. (**A(ii**)) Bar diagram representing MitoSOX staining in untreated, cholesterol treated cells with or without the pre-treatment of small molecule M1.Data are expressed as mean ± SEM of 3 independent experiments. Chol: cholesterol, M1: small molecule M1. (**B**) Measurement of mitochondrial membrane potential in control and cholesterol treated pancreatic beta cells with or without the pre-treatment of small molecule M1. The cells were stained with JC-1 dye and analyzed by flow cytometry. JC-1 monomers were detected in the FL1 channel (Green). JC-1 aggregates were detected in FL2 channel (Red) and the ratio of FL2/FL1 was calculated for the determination of mitochondrial membrane potential. The data is expressed as fold decrease of FL2/FL1 with respect to cholesterol untreated cells. (**C)** Untreated and cholesterol treated cells with or without M1 pre-treatment was stained with Mito-Tracker Red and imaged in Leica confocal laser scanning microscope (Germany) using Leica advanced fluorescence suite 2.6.3. Images are representative of three independent experiments (scale: 7.24 μm).

**Figure 5:**
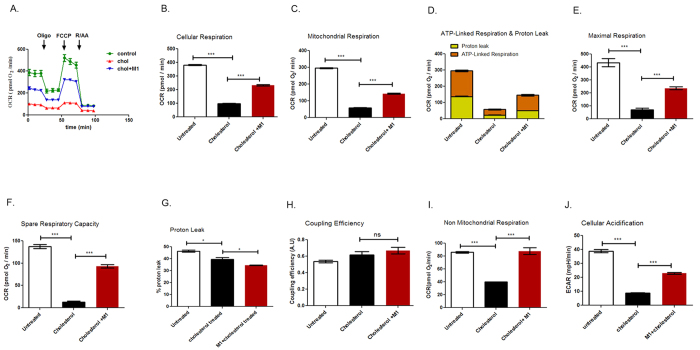
Real time respirometry analysis on the effect of cholesterol treatment of pancreatic beta cells. (**A**) Oxygen consumption rate at 10 mM glucose in presence and absence of inhibitors that interferes mitochondrial respiration is measured in control as well as cholesterol accumulated BRIN-BD11 pancreatic beta cells with or without the pre-treatment of small molecule M1. (**B)** Cellular respiration rate in untreated and cholesterol treated pancreatic beta cells with or without the pre-treatment of M1. (**C)** Mitochondrial respiration derived through subtraction of non-mitochondrial respiration rate from cellular respiration rate in untreated and cholesterol treated pancreatic beta cells with or without the pre-treatment of M1. (**D)** Mitochondrial respiration dissected as proton leak and ATP linked respiration in untreated and cholesterol treated pancreatic beta cells with or without the pre-treatment of M1. (**E)** Maximal respiration as obtained after the addition of chemical uncoupler FCCP and after subtraction of non-mitochondrial respiration rates in untreated and cholesterol exposed pancreatic beta cells with or without the pre-treatment of M1. (**F)** Spare Respiratory Capacity obtained by subtraction of basal respiration rate from maximal respiration rates in untreated and cholesterol exposed pancreatic beta cells with or without the pre-treatment of M1. (**G**) Proton leak derived through subtraction of ATP linked respiration from mitochondrial respiration and expressed as percentage of total mitochondrial respiration, in untreated and cholesterol treated pancreatic beta cells with or without the exposure of small molecule M1. (**H)** Coupling efficiency, calculated as fraction of oxygen consumption sensitive to oligomycin, in untreated and cholesterol treated pancreatic beta cells with or without the exposure of M1. (**I)** Non mitochondrial respiration determined by using electron transport chain inhibitors, rotenone and antimycin in untreated and cholesterol treated pancreatic beta cells with or without the exposure of M1. (**J)** Extracellular acidification rate which gives an approximate estimation of glycolysis in untreated and cholesterol treated pancreatic beta cells with or without the pre-treatment of M1. All data are normalized for protein content , expressed as mean ± SEM of 3 independent experiments and presented as pmol O_2_/min (***P < 0.001 *t*-tests (unpaired)), ns: not significant. Chol: cholesterol.

GLP-1R potentiation of insulin secretion in primarily driven by cAMP generation, however, recent studies by Hodson *et al*.^27^ described GLP-1R mediated modulation of a metabolically coupled sub-network in pancreatic beta cells which increases cytosolic ATP levels. In the present study we explored whether cellular respiration is perturbed due to the increase in total cholesterol in cultured pancreatic beta cells. The cholesterol accumulation enhances the mitochondrial reactive oxygen species (mito-ROS) by 2.33 ± 0.29 fold (Fig. 4A(iii)), decreases mitochondrial hyperpolarization to 0.29 ± 0.05 fold (Fig. 4B) and alters mitochondrial architecture (Fig. 5C). The Western blot data followed by densitometric analysis reveals that Calnexin which is rough ER–MAM biomarker^28^,^29^ is reduced by 0.45 fold in mitochondrial fraction due to cholesterol accumulation in pancreatic beta cells (supplementary Fig. 1B(i)). Since Mitofusin -2 (Mfn-2) is a key component of ER-mitochondria contact sites^30^ we treated the cells with (E)-4-Chloro-2-(1-(2-(2,4,6-trichlorophenyl) hydrazono) ethyl) phenol (small molecule M1) which was known to promote mitochondrial elongation in both Mitofusin-1 and Mitofusin -2 knock-out fibroblasts (Wang and Schultz 2011^31^, methods, supplementary Fig. 2). Our data reveals that pre-treating BRIN-BD11 pancreatic beta cells with small molecule M1 decreases mito ROS to 1.0 ± 0.44 fold (Fig. 4A(i,ii)), enhances mitochondrial membrane potential from 0.29 ± 0.05 fold to 0.5 ± 0.07 fold (Fig. 4B) and restores mitochondrial architecture (Fig. 5C). However, we do not observe any change in Calnexin-Cytochrome oxidase (Cox IV) ratio on M1 pre-treatment in cholesterol enriched pancreatic beta cells (supplementary Fig. 1B(ii)) indicating that small molecule M1 may not have any direct role in preserving endoplasmic reticulum-mitochondria contact which has important role in regulating mitochondrial function.

Since we observed alteration of mitochondrial architecture on cholesterol enrichment in pancreatic beta cells we enquired whether the expression of fusion and fission genes which preserves mitochondrial architecture is affected. As our data shows, exposure to cholesterol for 12 h increases Mfn-1 by 3.07 ± 1.35 fold with no major alteration in expression of Mfn-2, Opa-1, Drp-1, Fis-1or MTP-18 (supplementary Fig. 3). The rationale behind this alteration of mitochondrial architecture is presently unclear. We believe that the alteration might reflect on the compromise in the functional status of the organelle as has been reported in biopsy samples of pancreatic beta cells isolated from type 2 diabetic patients^32^.

### Impairment of oxygen consumption rate in pancreatic beta cells due to cholesterol exposure and its prevention by small molecule M1

We next explored whether exposure to 160 μM cholesterol for 12 h impairs the metabolic function of mitochondria which has important role in insulin granule exocytosis. Incubation of MIN6 cells with 160 μM soluble cholesterol for 24 h and 48 h has been reported to generate oxidative stress^33^. In the *in vivo* context, Caveolin-1 knockout mice shows that accumulation of the free cholesterol in mitochondrial membrane^34^ reduces its fluidity, attenuates the efficiency of the respiratory chain and increases mitochondrial ROS^35^. In the present study, we carried out a real time respirometry analysis of control and cholesterol enriched pancreatic beta cells using XFe 24 analyzer (SeaHorse Bioscience). Figure 5A provides the graphical description of cellular respiration and respiration after the interference of mitochondrial pathways with specific inhibitors. As the data shows, basal oxygen consumption rate (OCR) in presence of 10 mM glucose was decreased from 381.48 ± 1.76 to 96.87 ± 2.38 pmol/min in cholesterol enriched pancreatic beta cells. Small molecule M1 pre-treated cells experienced a much less decrease of OCR to 233.1 ± 3.88 pmol/min on cholesterol accumulation (Fig. 5B). Figure 5C shows the effect of cholesterol accumulation on mitochondrial respiration in pancreatic beta cells. Cholesterol enrichment decreases mitochondrial respiration from 259.49 ± 4.76 to 57.73 ± 3.89 pmol/min. In case of small molecule M1 pre-treated cells, cholesterol accumulation reduces mitochondrial respiration to 143.16 ± 6.60 pmol/min which is significantly higher (p < 0.001) compared to cholesterol accumulated pancreatic beta cells. In concert, ATP-linked respiration which was attenuated from 158.35 ± 9.34 pmol/min to 34.92 ± 4.21 pmol/min is enhanced to 95.96 ± 10.11 pmol/min on small molecule M1 pre-treatment (Fig. 5D). There is also a profound impediment of maximal respiration (Fig. 5E) and spare respiratory activity (Fig. 5F) which was reduced from 433.54 ± 43.76 pmol/min to 70.76 ± 26.91 pmol/min and from 137.11 ± 6.31 pmol/min to 13.03 ± 3,82 pmol/min respectively due to cholesterol accumulation. Small molecule M1 pre-treatment significantly prevents this decrease; maximal respiration is reduced to 236.17 ± 13.17 pmol/min and spare respiratory activity is reduced to 93.01 ± 6.39 pmol/min which is respectively 3.34 fold and 5.37 fold higher in comparison to small molecule M1 untreated cholesterol accumulated pancreatic beta cells. The data thus highlights the role of small molecule M1 in prevention of attenuation of mitochondrial respiration in pathophysiological context of cholesterol accumulation.

Respirometry analysis by Wikstrom *et al*.^36^ reported pancreatic islet mitochondria to be highly uncoupled in nature; in our present study with BRIN-BD11 pancreatic beta cell mitochondria, the proton leak is 46.41 ± 1.48% of the total mitochondrial respiration which is in concert with the previous observation in pancreatic islets (Fig. 5D). Cholesterol accumulation in mitochondria decreases the proton leak to 39.51 ± 2.5% which was found to be statistically significant (p < 0.05) (Fig. 5G). The mitochondrial coupling efficiency which is measured as fraction of mitochondrial oxygen consumption that is sensitive to oligomycin also remained unaltered in untreated and cholesterol enriched pancreatic beta cells (Fig. 5H). The data thus indicates that cholesterol enrichment does not impact the efficiency of protein complex in the inner membrane that generates the proton motive force; rather it may impact substrate availability and /or shuttle function consequently affecting the mitochondrial respiration.

### Impairment of non-mitochondrial respiration and extracellular acidification rate (ECAR) on cholesterol enrichment and its prevention by small molecule M1

We next determined whether cholesterol exposure to pancreatic beta cells modulates non-mitochondrial respiration and extracellular acidification rate which serves as an approximate index for glycolysis. As Fig. 5I reveals, cholesterol enrichment reduces non-mitochondrial respiration from 85.99 ± 2.22 pmol/min to 39.87 ± 0.89 pmol/min. M1 pre-treatment prevents this reduction and preserves OCR at 87.22 ± 9.24 pmol/min. Similarly, extracellular acidification rate, which was reduced from 38.83 ± 1.99 mpH/min to 8.79 ± 0.56 mpH/min due to cholesterol enrichment exhibited lesser attenuation (22.93 ± 1.00 mpH/min) (Fig. 5J) on pre-treatment with small molecule M1 highlighting its hitherto unknown role in stimulating non-mitochondrial respiration in cholesterol enriched pancreatic beta cells.

### Small molecule M1 restores GSIS in cholesterol treated pancreatic beta cells

**Figure 6:**
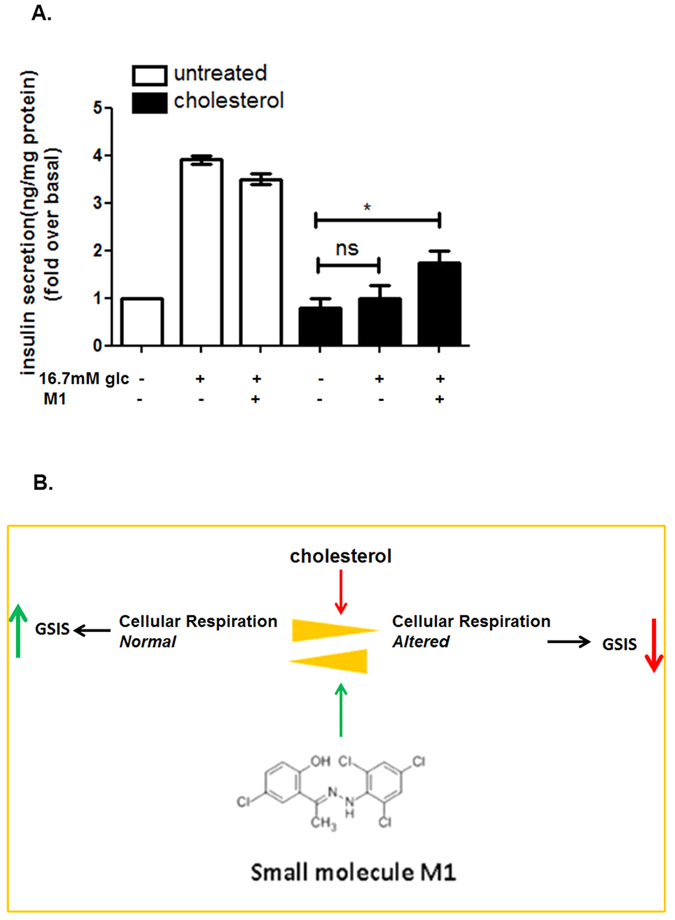
Small molecule M1 mediated prevention of loss of GSIS in cholesterol enriched pancreatic beta cells. **(A)** Pre-treatment with small molecule M1 prevents loss of GSIS in cholesterol enriched pancreatic beta cells. Chol: Cholesterol. (**B**) Proposed model of M1 mediated preservation of cellular respiration which prevents the loss of GSIS in cholesterol exposed pancreatic beta cells.

Our present study shows that small molecule M1 stimulates cellular respiration, maintains the mitochondrial architecture, enhances mitochondrial membrane potential and reduces mitochondrial ROS. We further explored whether these effects culminate in the potentiation of GSIS in cholesterol enriched pancreatic beta cells. As Fig. 6A reveals, small molecule M1 mediated stimulation of cellular respiration, prevention of alteration of mitochondrial architecture and function stimulates GSIS by 2.32 ± 0.39 fold which is abolished on cholesterol enrichment in pancreatic beta cells. The data also shows that M1 does not potentiate GSIS in untreated pancreatic beta cells indicating at the functional relevance of the molecule in maintaining GSIS under the pathophysiological condition of cholesterol accumulation.

## Discussion

Impairment of GSIS due to cholesterol accumulation in pancreatic beta cells has been described in various mouse models^13^,^15^ as well as in patients harboring autosomal recessive mutations in ABCA1 gene^14^. In our present study we adopted machine learning approach using support vector machines to explore the impact of cholesterol on fasting blood sugar in Indian population. Our study reveals that serum cholesterol has a higher linear impact on fasting blood sugar than serum triglycerides indicating at the importance of cholesterol homeostasis on the progression of type 2 diabetes. We evaluated the macronutrient consumption of the population analysed in our study and accordingly designed the experimental diet to induce hypercholesterolemia in experimental rats. The experimental diet design is in concert with the published literature which describes the mechanism by which dietary cholesterol and fat modulates the lipid parameters in animal model as well as in human subjects^37^,^38^,^39^. Dietary cholesterol is known to expand sterol pools in liver and decrease LDL receptors (LDLR) which are involved in scavenging LDL cholesterol from blood. Long chain fatty acids further suppress the hepatic LDLR expression while unsaturated fatty acids reverse the effect^39^, Recently, Abreu *et al*.^40^ reported that feeding Fischer rats with hypercholesterolemic diet comprising of 25% soyabean oil +1% cholesterol for 8 weeks enhanced serum cholesterol but has no effect on blood glucose. In our present study we fed Sprague-Dawley rats with experimental diet containing 20% peanut oil, standard nutrients, 1% cholesterol with Fructose as source of carbohydrate for three months. The animals showed hypercholesterolaemia, hyperinsulinemia with modest elevation of blood glucose level and without any increase in body weight or change in adipose tissue mass. Significantly, the animal group fed on experimental diet containing 20% Peanut oil and standard nutrients with starch or fructose as a source of carbohydrate does not show any increase in serum cholesterol, serum triglycerides, serum insulin or blood glucose. The pancreatic islets of the rat on hypercholesterolemic diet revealed a significant increase in total cholesterol. To explore the relationship of cholesterol accumulation with insulin exocytosis we treated cultured BRIN-BD11 pancreatic beta cell with 160 μM cholesterol for 1 h or 12 h. The concentration of soluble cholesterol and the time period of incubation was selected so that apoptosis was not induced as was evident from that fact that both 1 h and 12 h incubation with soluble cholesterol causes significant increase in cAMP generation which is known to inhibit apoptosis through CREB mediated induction of IRS2^23^ and through stimulation of IAP2^24^. The data is in concert with the cell survival assays which also shows no cell death on treatment of BRIN-BD11 cells with 160 μM cholesterol for 12 h. While our manuscript was under submission, Carasco-Pozzo *et al*.^41^ reported that 3–4 di-hydroxy phenyl acetic acid, a microbiota derived metabolite of Quercetin, prevents cholesterol induced apoptosis of MIN6 pancreatic beta cells. The difference in our observation on cell death is most likely due to the time period of incubation and concentration of soluble cholesterol which was double in comparison to the concentration of soluble cholesterol and time period of treatment followed in our experiments.

The data presented in this study described the complexity of the modulation of GSIS due to enhancement in total cellular cholesterol. In fact, the cholesterol enrichment has severe repercussions on mitochondrial function; as our data reveals, cholesterol enrichment increases mitochondrial ROS, decreases mitochondrial hyperpolarization and impedes cellular respiration thus profoundly affecting cellular metabolic function.

Insulin secretion from pancreatic beta cells is programmed to respond to the fluctuating blood glucose levels and this coupling of glucose sensing to insulin secretion is maintained by glucokinase through the regulation of the inflow of glucose carbons to mitochondria^11^. Cholesterol accumulation has been reported to keep glucokinase in membrane bound inactive state^8^ thus compromising the flux of glucose carbon to mitochondria. We observed reduced non-mitochondrial respiration and ECAR which is proportional to glycolysis in cholesterol accumulated pancreatic beta cells. The data indicates that reduction of the availability of glucose carbons might be one of the possibility for the alteration of mitochondrial function in cholesterol accumulated pancreatic beta cells.

Cholesterol enrichment in pancreatic beta cells has been shown in this study to alter the shape of mitochondria from filamentous to knot shaped structure. We also observed diminished expression of calnexin which is ER-MAM marker^28^,^29^. The data indicates at the reduced contact between ER and mitochondria in cholesterol accumulated pancreatic beta cells which may have impact on mitochondrial function. Impact of MAM on mitochondrial metabolism in context of regulation of GSIS is incompletely understood at the present moment; in-depth understanding on the role of MAM in regulating mitochondrial signaling would enhance the present knowledge on the patho-physiological mechanism by which GSIS is compromised in cholesterol enriched pancreatic beta cells.

Architecture of mitochondria has been considered as a useful determinant of the metabolic conditions inside the organelle^42^. Anello *et al*. described the grossly altered ultrastructure and reduced functional activity of mitochondria isolated from type 2 diabetic patients^32^. This alteration in architecture compromises dynamic mitochondrial networks which are modulated by continuous fission and fusion, the process being regulated by the phospholipid composition at the fission sites^43^ as well as by the members of dynamin like GTPases^42^. Role of mitochondrial fission/fusion proteins in regulating GSIS has been essentially documented both *in vivo* and *in vitro* studies^44^, however, their association with mitochondrial membrane phospholipids in maintaining mitochondrial integrity and function is presently unknown. Recently, Wang *et al*. identified a small molecule M1 that dose dependently induces the elongation of mitochondria in Mitofusin1 (Mfn1) and Mitofusin 2 (Mfn-2) ablated mouse embryonic fibroblasts^31^. Since Mfn2 is a major constituent of MAM, the contact site between ER and mitochondria, which plays role in cholesterol metabolism^30^, we treated the cells with small molecule M1 to explore its role in regulating mitochondrial function in cholesterol accumulated pancreatic beta cells. As our data reveals, the small molecule M1 decreases mitochondrial ROS, restores organelle architecture, stimulates mitochondrial respiration and consequently enhances mitochondrial membrane potential in cultured BRIN-BD11 pancreatic beta cells. However, contrary to our expectations, the western blotting data followed by densitometry reveals no change in calnexin: Cox IV ratio on small molecule M1 pre-treatment. The data indicates that the mitochondrial fusion promoter M1 may not have any role in preserving ER-mitochondria contact which was compromised due to cholesterol accumulation in pancreatic beta cells.

Our study revealed that small molecule M1 pre-treatment prevents the reduction of non -mitochondrial respiration as well as ECAR in cholesterol enriched pancreatic beta cells.; Glucokinase, in its inactive form , has been reported to be associated with insulin secretory granules^45^. Since insulin secretory granules are one of the sites of intracellular cholesterol accumulation^26^ and cholesterol accumulation has been reported to stabilize glucokinase at insulin granules in membrane bound inactive state^8^, there is a distinct possibility for a novel role of small molecule M1 to shift the equilibrium of glucokinase from inactive membrane bound state to soluble cytoplasmic active state. However, a detailed work has to be carried out in this regard to decipher the role of small molecule M1 in potentiating glycolysis in cholesterol enriched cultured pancreatic beta cells.

The data presented in our study thus clearly shows that reduction in cellular respiration due to accumulation of cellular cholesterol blunts GSIS in pancreatic beta cells. Treatment with small molecule M1 though significantly enhanced GSIS could not restore it to the normal level indicating that cholesterol treatment impaired insulin exocytosis at multiple levels of the secretory pathway.

In summary, the document describes cholesterol accumulation as a major cause of impediment of cellular respiration in pancreatic beta cells that attenuates GSIS. M1 has been previously reported to alleviate the neuronal toxicity in cell based model of Parkinson’s disease pathogenesis^31^. Our results, as summarized in Fig. 6B furnished a novel role of M1 in preserving the mitochondrial function and promoting cellular respiration thereby stimulating GSIS in cholesterol accumulated pancreatic beta cells. The observation may open up a novel therapeutic window for ameliorating hyperglycemia in patients having enhanced serum cholesterol and LDL as co-morbidities which aggravate the pathophysiology of patients suffering from type 2 diabetes.

## Methods

### Animals

Twelve weanling male Sprague Dawley rats were obtained from animal house facility of National Institute of Nutrition (Hyderabad, India). All the procedures involved in animal experimentation were carried out in accordance with Institutional Animal Ethical Committee following international standards. All experimental protocols were approved by the Institutional Animal Ethical Committee of National Institute of Nutrition (Hyderabad, India) and the experiments were carried out following the approved protocols. Animals were housed in laboratory cages at 23 °C under a 12 h light/dark cycle. Rats were divided equally into three groups and fed with either experimental diet containing 20% Peanut oil, standard nutrients with starch or fructose as the source of carbohydrate or diet containing 20% Peanut oil, standard nutrients and 1% cholesterol with fructose as the source of carbohydrate as described in Table 1. All the animals were fed on respective experimental diets and at the end of 3 months, fasting blood was collected and plasma was separated and analyzed for lipids, glucose and insulin. Plasma glucose, triglycerides and cholesterol were estimated using enzymatic methods (Biosystems, Spain) and insulin by ELISA using kit from Marcodia, Spain. Total cholesterol from islets was estimated by enzymatic kit method (Biosystem Spain).

### Analysis of Fasting Blood Sugar, Insulin, Total cholesterol, serum triglycerides, HDL and LDL levels in human subjects

All experimental protocols were approved by Institutional Ethics Committee (IEC), University of Hyderabad, Hyderabad 500046, India, following international standards and informed consent was obtained from all human subjects. Methods of human sampling were carried out in accordance with the approved guidelines of the committee.

Blood was taken from the normal volunteers and type 2 diabetic patients after overnight fasting following ethical clearance of the institute and with patient’s consent. The Fasting Blood glucose level was measured with a glucose oxidase kit. The serum insulin level was measured with Insulin Radio Immune Assay kit. Total cholesterol, triglycerides, and HDL and LDL levels were measured following routine laboratory procedures. The data set comprises of normal volunteers and type 2 diabetic patients (n = 144), each data set being associated with individual parameters of Fasting Blood Sugar (FS), Insulin (In), Family History of Known Diabetes (FH), Age, Total Cholesterol (TC), Triglyceride (TGL), HDL (High Density Lipoprotein) and LDL. The data analysis was carried out as per machine learning parlance in Rapidminer™ Studio and the regression machine was constructed using support vector algorithm. Statistical estimation for optimal coefficients was established by cross validation with fold size (=10), the technique takes into account statistical sampling error (estimated by RMSE and SE) as well as model specification error that is missed by the conventional statistical method. To execute a more rigorous estimation of statistical significance we adopted evolutionary computation approach and applied a much tougher model validation technique such as LOO (Leave One Out) to confirm the linearity of the relationship and determine the coefficients of the predictors.

### Synthesis of 4-chloro-2-(1-(2-(2,4,6-trichlorophenyl) hydrazono) ethyl) phenol (M1)

All reactions were carried out in oven-dried glassware under Nitrogen atmosphere. Thin layer chromatography (TLC) was carried out on aluminium sheets coated with silica gel 60 F254 (Merck, 1.05554) and the spots were visualized with UV light at 254 nm. Flash column chromatography was performed using silica gel (Merck, 60A, 230–400 Mesh). Commercially available reagents were used as supplied and some of them were distilled before use. Proton (^1^H) and carbon (^13^C) NMR spectra were recorded in CDCl_3_ or DMSO-*d*_*6*_ on a Varian 400 MHz spectrometer with the signals arising from the residual solvent being used as the internal standards. LR-MS and LC-MS data were recorded on an Agilent 1200 Series liquid chromatography module hyphenated to a 6430 Triple Quad LC/MS system. Synthetic route to 4-chloro-2-(1-(2-(2,4,6-trichlorophenyl)hydrazono)ethyl) phenol (M1) is shown in supplementary Fig. 2.

### Preparation of 4-chlorophenyl acetate (2)

To a stirred solution of AlCl_3_ (31.0 g, 0.233 moles) in dichloromethane at 0 °C, acetyl chloride (15.3 g, 0.194 moles) was added and stirred for 15 min at 0 °C. A solution of 4-Chlorophenol (1) (20.0 g, 0.156 moles) in dichloromethane was added to the reaction mixture over a period of 15 min at 0 °C, and was allowed to warm to room temperature and stirred for 3.5 h. After completion of the reaction, the mixture was quenched with 1N aq. HCl and the organic layer was washed with saturated aqueous NaHCO3, followed by brine. The organic layer was dried (anhydrous Na_2_SO_4_) and concentrated *in vacuo* to obtain 4-chlorophenyl acetate **(2)** (18.5 g, 70%) as colorless liquid.^1^H NMR (400 MHz, CDCl_3_): δ 7.33 (m, 2H), 7.02 (m, 2H), 2.29 (s, 3H); Mass: *m/z* = 169 [M - H] ^+^

### Preparation of 1-(5-chloro-2-hydroxyphenyl) ethanone (3)

The mixture of 4-chlorophenyl acetate 2 (18.5 g, 0.108 moles) and triflic anhydride (148 mL) was heated to 85 °C and stirred for 16 h. The progress of the reaction was monitored by TLC. After completion of the reaction, the reaction mixture was cooled to 0 °C and quenched with saturated NaHCO_3_ solution. The aqueous solution was extracted with DCM (2 × 100 mL) and washed with brine solution, the combined organic extracts were dried over anhydrous Na_2_SO_4_ and concentrated under reduced pressure to furnish 1-(5-chloro-2-hydroxyphenyl)ethanone 3 (12 g, 65%) as a white solid. ^1^H NMR (400 MHz, CDCl_3_): δ 12.13 (s, 1H), 7.68 (d, 1H, *J* = 3.2 Hz), 7.41 (dd, 1H, *J* = 9.2 Hz, 2.4 Hz), 6.94 (d, 1H, *J* = 9.6 Hz); Mass: *m/z* = 169 [M - H]^+^

### Preparation of 4-chloro-2-(1-(2-(2,4,6-trichlorophenyl) hydrazono) ethyl) phenol (M1)

A mixture of 1-(5-chloro-2-hydroxyphenyl) ethanone **(3)** (12 g, 0.0703 moles), (2,4, 6-trichlorophenyl)hydrazine (16.37 g, 0.077 moles), EtOH (60 mL) and catalytic quantity of AcOH were heated to reflux temperature and stirred for 1 h. The progress of the reaction was monitored by TLC, after completion of the reaction, the reaction mixture was cooled to 0 °C and the obtained solid was filtered and washed with chilled EtOH. The compound was dried under vacuum for 1 h to furnish 4-chloro-2-(1-(2-(2,4,6 trichlorophenyl)hydrazono)ethyl)phenol (M1) (15.37 g, 60%) as an off white solid.^1^H NMR (400 MHz, DMSO-d6): δ 12.01 (s, 1H), 8.64 (s, 1H), 7.81 (s, 1H), 7.61 (d, 1H, *J* = 2.4 Hz), 7.30 (dd, 1H, *J* = 8.8 Hz, 2.8 Hz), 6.90 (d, 1H, *J* = 8.8 Hz); ^13^C NMR (100 MHz, DMSO-d6): δ 156.59, 150.47, 138.20,130.40, 130.05, 129.86, 129.31, 127.46, 122.85, 122.23, 118.77, 13.65; Mass: *m/z* = 365 [M + H]^+^; LCMS purity 99.6%

### Cell Culture and treatment

BRIN-BD11 cells were cultured at 37 °C with 5% CO_2_ in RPMI media supplemented with 10% heat inactivated Fetal Bovine Serum (FBS) (Gibco, cat No: 10082-147, heat inactivated, US origin), 1 mM Sodium pyruvate, 10 μg/ml Gentamicin, 100 units/ml Penicillin and 100 μg/ml Streptomycin.

Cholesterol treatment in BRIN-BD11 cells were carried out following the method of Lu *et al*.^33^ with modifications. 250X Cholesterol lipid concentrate (CLC) (cholesterol ∼ 7 mM) was purchased from GIBCO (Grand Island, NY, USA). The CLC was diluted at different concentrations in growth medium and was added to BRIN-BD11 cells at different time points. In case of small molecule M1 pretreatment, cells were first treated with small molecule M1 for 12 h and then cholesterol (160 μM) was added to the media for another 12 h prior to the assay being carried out.

### CRE Luciferase Reporter assay

Receptor mediated signaling was assessed by Luciferase reporter assay following the method of Fortin *et al*. with modifications^46^. In brief, the cells were grown in 70 mm dish until they attain 70% confluence. A cAMP responsive element-luciferase reporter plasmid (CRE_6X_-luc), which is a kind gift of Prof Richard Day at Indiana University, and beta galactosidase plasmid were transfected in 1:1 ratio using Turbofect transfection reagent following manufacturer’s protocol. Soluble cholesterol treatment at indicated concentrations was carried out for different time period in complete media 48 hours after the transfection following which the cells were treated with100 nM Exendin-4 for 4 h. The media was then aspirated, cells were lysed and luciferase activity was measured using Steady lite plus reagent (Perkin Elmer Life and Analytical Science, Waltham, MA). To enable the correction for the inter-well variability of transfection, beta galactosidase assay was performed by the addition of 2-nitrophenyl-beta galacto pyranoside (Sigma). After incubation for 15 min at 37 °C, substrate cleavage was quantified by measuring the optical density at 405 nm in ELISA plate reader (Perkin Elmer, USA) and the corresponding values were used to normalize luciferase activity.

### Cell Viability assay

Cell viability was measured quantitatively by assessing MTT dye reduction. Cells were seeded at 25000 cells/well in 96 well plates, allowed to grow for 24 h and cell viability was assessed after treatment with 160 μM soluble cholesterol at different time points in presence and absence of small molecule M1. At the end of incubation period, MTT dye (5 mg/ml) was added at 25 μL per 100 μL media for 2 h. The formazan crystals were solubilized by 100 μL solution of 20% SDS prepared in 50% DMF solution. The absorbance was measured at 570 nm using microplate reader (Perkin Elmer Victor multi label counter).

### Analysis of mitochondrial membrane potential (∆ψM)

Mitochondrial membrane potential was determined by JC-1 staining kit (Cat. no. CS0390, Sigma). 1 × 10^6^ BRIN-BD11 cells were seeded in 6 well plates and cultured overnight in complete medium. Untreated cells and cells treated with 160 μM cholesterol for 12 h in presence and absence of small molecule M1 were assessed. After treatment, the cells were washed with PBS (Phosphate Buffer Saline) and stained with 2.5 μM of JC-1 in 1x JC-1 staining buffer. Cells were incubated for 20 minutes at 37 °C in a humidified atmosphere containing 5% CO_2_ and after staining washed with ice-cold 1x JC-1 Staining Buffer. The cells were then resuspended in 0.5 ml of the same buffer and the analysis of the samples was performed using a FACSCalibur, (BD Bioscience, San Jose, CA) flow cytometry system. JC-1 monomers were detected in the FL1 channel (green). JC-1 aggregates were detected in FL2 channel (red) and the ratio of FL2/FL1 was calculated for determination of mitochondrial membrane potential.

### Analysis of mitochondrial ROS

BRIN-BD11 cells at a density of 1 × 10^6^ cells per well were seeded in 6 well plates and cultured overnight in complete medium. Untreated and cells treated with 160 μM cholesterol with or without small molecule M1 pre-treatment were stained for 30 min with fluorogenic dye MitoSOX™ Red (Molecular Probes) for mitochondrial ROS measurement following manufacturer’s instructions. The flowcytometric analysis was carried out on live cells, using FACSCalibur, (BD Bioscience, San Jose, CA).

### Insulin secretion assay

Insulin secretion studies from BRIN-BD11cells were carried out following the method of Irwin *et al*.^47^ with modifications, using a Millipore Rat/Mouse Insulin ELISA kit (cat. no. EZRMI-13K). The cells were seeded in 24 well plates at a density of 1 × 10^5^ cells/well and cultured overnight in complete medium. The cells were washed with PBS and pre-treated with 300 μL of Krebs-Ringer bicarbonate buffer [115 mM NaCl, 4.7 mM KCl, 1.28 mM CaCl_2_, 1.2 mM KH_2_PO_4_, 1.2 mM MgSO_4_, 10 mM NaHCO_3_. 0.1% (BSA, pH 7.4] for 60 min after which the agonist was added in presence or absence of 16.7 mM glucose. Ten microliters of cell supernatant was used for the ELISA. The insulin release was measured as nanograms/mg protein. Insulin release at 0.1 mM glucose was considered as basal insulin secretion.

### Immunofluorescence microscopy

BRIN-BD11 cells were plated in six-well plates containing 25-mm-diameter glass coverslips and cultured overnight. After 12 h of indicated cholesterol treatment with or without the pre-treatment with small molecule M1, complete media was removed, cells were washed with PBS and stained with 200 nM Mito-Tracker Red (M7512) in complete medium at 37 °C for 30 min, washed again with PBS and fixed with 4% paraformaldehyde. The coverslips were then mounted in Vectashield (Vector Laboratories) and imaged in Leica confocal laser scanning microscope (Germany) using Leica advanced fluorescence suite 2.6.3. For GLP-1R internalization study,BRIN-BD11 cells were transfected with GLP-1R-GFP plasmid using Turbofect transfection reagent and plated in six-well plates containing 25-mm-diameter glass coverslips. Sixty hours after transfection, cells in coverslips were incubated with GLP-1-Tmr (GLP-1- Tetra methyl rhodamine) (100 nM) in 200 μl of Krebs-HEPES buffer for 60 min at 4 °C in the dark. The cells were then washed in PBS and incubated at 37 °C for the desired time period in complete medium, after which they were fixed in 4% paraformaldehyde, mounted in Vectashield mounting medium (Vector Laboratories), and imaged using a Leica confocal laser scanning microscope (Germany).

### Estimation of total cholesterol

The cells were seeded in 60 mm dish plates at a density of 1.5*10^6^ cells/dish and cultured overnight in complete medium. The cells were treated with 160 μM cholesterol for indicated time. The amount of total cholesterol in BRIN-BD11cells was measured using a Sigma cholesterol quantitation Kit (cat. no. MAK043). Briefly, the cells were trypsinized and the cholesterol was extracted with 200 μL of chloroform: isopropanol: Igepal CA-630 (octyl phenoxy poly ethoxy ethanol) in a ratio of 7:11:0.1 in a micro homogenizer. The samples were then centrifuged at 13,000 g for 10 minutes to remove insoluble material. The organic phase was transferred to a new tube and air dried at 50 °C to remove chloroform. The pellet was discarded and the samples were dried under vacuum for 30 minutes to remove any residual organic solvent. Finally the dried lipids were dissolved with 200 μL of the cholesterol assay buffer. The concentration of total cholesterol present in the sample was determined by a coupled enzyme assay following manufacturer’s instructions. The colorimetric product which was generated proportional to the cholesterol present in the sample was measured at 570 nm. For total cholesterol estimation, Cholesterol Esterase was added in the reaction mixture which hydrolyzes cholesteryl esters to cholesterol. In the presence of Cholesterol Esterase, the assay detects total cholesterol, both free cholesterol and cholesteryl esters.

### Estimation of cholesterol from subcellular fraction which comprises of mitochondria, secretory granules and endosomes

Isolation of mitochondria from BRIN-BD11 pancreatic beta cells was carried out following established protocols. Briefly, 1 × 10^7^ BRIN-BD11cells were grown in 150 mm dishes and treated with 160 μM cholesterol with or without the presence of 20 μM small molecule M1. Cells were harvested from 150 mm dishes and washed with isolation buffer (10 mM HEPES-KOH, pH7.2 containing 1.5 mM MgCl2, 1 mM EDTA, 1 mM EGTA, 0.21 M sucrose, 70 mM mannitol and protease inhibitors). 1 × 10^7^ cells were re suspended in 10 ml isolation buffer and kept on ice for 60 min with frequent tapping. The cellular suspension was homogenized with a glass homogenizer with 60 times up and down passes of the pestle. The homogenate was centrifuged at 2700 rpm for 10 min at 4 °C to remove the nuclei and intact cells, and the supernatant was centrifuged at 9000 rpm for 15 min at 4 °C. The resulting supernatant (cytosolic fraction) was removed while the pellet was collected for cholesterol estimation. The purity of the fraction was measured in western blot; Cox IV antibody (Santa Cruz Biotechnology, sc58438) was used as mitochondrial marker, Calnexin (H-70) (Santa Cruz Biotechnology, sc11397) was used as marker for ER-MAM and GAPDH (Imgenex IMG 6665A) was used as marker for cytosolic fractions. Ponceau S stained PVDF membrane (supplementary Fig. 1b(iii)) provided the evidence for equal loading. Total cholesterol was estimated as described in the previous section.

### Real time respirometry to measure oxygen consumption rate (OCR) and extracellular acidification rate (ECAR)

The cells were seeded in an XFe^24^ 24-well cell culture microplate at 20,000 cells/well in 500 μL of growth medium and incubated overnight at 37 °C in CO_2_, in presence and absence of 160 μM cholesterol and with or without pre-treatment with small molecule M1. The cells were washed with assay medium comprising 114 mM NaCl, 4.7 mM KCl, 1.2 mM KH_2_PO_4_, 1.16 mM MgSO_4_, 20 mM HEPES pH 7.4, 2.5 mM CaCl_2_ and supplemented with 10 mM glucose and incubated in this medium for 60 min at 37 °C in air. Plates were transferred to a Seahorse Bioscience XFe^24^ extracellular flux analyzer (controlled at 37 °C) and subjected to an equilibration period. To inject Oligomycin, FCCP & Rotenone/Antimycin (Mito stress kit constituents) we have followed a constant concentration/variable loading strategy as per the manufacturers’ protocol. The concentration of Oligomycin and FCCP was determined through titration. One assay cycle comprised of 1-min mix, 2-min wait and 3-min measure period. After measuring basal OCR for 3 cycles, Oligomycin (1 μM) was added to inhibit the ATP synthase and thus determine the proportion of respiration used to generate ATP synthesis. After 4 further assay cycles, carbonyl cyanide-4-(trifluoro methoxy) phenyl hydrazone FCCP (1 μM) was added to determine maximal respiration by mitochondria by uncoupling ATP synthesis from electron transport. After another 4 assay cycles Rotenone (0.5 μM) plus Antimycin (0.5 μM) was added to measure non- mitochondrial respiratory rate. Three readouts were measured for basal & each inhibitor injection. Extracellular acidification rate was measured simultaneously to OCR.

### Real-time RT-PCR

Total RNA was extracted from cholesterol treated and untreated BRIN-BD11 cells using TRIzol reagent (Life Technologies) and 1 μg of total RNA was used to synthesize cDNA using Superscript III First-Strand cDNA synthesis kit. Quantitative real-time PCR was performed in the Step One -Plus Real -Time PCR system using kapa sybr fast universal master mix (Kapa Biosystems). The relative abundance of the mRNAs was measured using 2^−ΔΔCT ^48 with GAPDH mRNA as invariant reference.

### Western blotting for PKA substrate

BRIN-BD11 cells were treated with soluble cholesterol (160 μM) in serum free medium for 12 hours followed by treatment with KRB medium for 60 min. After the treatment, the cells were stimulated with 100 nM Ex-4 for 30 min and were lysed in cell lysis buffer containing protease inhibitor (Sigma) and phosphatase inhibitor (EMD Millipore). Fifty micrograms of the cell extracts were subjected to Western blot analysis using phospho-PKA substrate antibody (cat. no. 9621, Cell Signaling Technology) following standard procedures. Actin immunoblot was used as a loading control.

### Statistical analysis

The data are presented as mean ± SEM. Statistical significance (*P* < 0.05) was assessed by Student’s *t*-test (unpaired) and one way ANOVA.

## Additional Information

**How to cite this article**: Asalla, S. *et al*. Restoring Mitochondrial Function: A Small Molecule-mediated Approach to Enhance Glucose Stimulated Insulin Secretion in Cholesterol Accumulated Pancreatic beta cells. *Sci. Rep.* **6**, 27513; doi: 10.1038/srep27513 (2016).

## Supplementary Material

Supplementary Information

## Figures and Tables

**Table 1:** Composition of the diet used in the study (gram/100 gram diet).

Nutrients	Experimental Diet1	Experimental Diet 2	Experimental Diet 3
Starch	44.5	0	0
Fructose	0	44.5	44.5
Casein	25	25	25
Peanut oil	20	20	20
Cellulose	5	5	5
Salt mixture	4	4	4
Vitamin mixture	1	1	1
Cysteine	0.3	0.3	0.3
Choline Chloride	0.2	0.2	0.2
Cholesterol	0	0	1

**Note:** Salt and vitamin mixtures were prepared according to AIN-93 (Reeves *et al*., Journal of Nutrition, 123 (1993)^49^.

**Table 2:** Fasting Blood sugar, insulin, lipids and total cholesterol in pancreatic islets.

	Experimental diet 1 (Starch + 20% peanut oil + regular nutrients)	Experimental diet 2 (Fructose + 20%peanut oil + regular nutrients	Experimental diet 3 (Fructose + 20%peanut oil+ regular nutrients + 1% cholesterol
Glucose (mg/dl)	96.7 ± 6.4^ab^	91.2 ± 5.0^a^	109.5 ± 5.1^b^
Insulin (μU/ml)	15.8 ± 2.2^a^	17.8 ± 2.5^a^	28.4 ± 2.8^b^
Triglycerides (mg/dl)	27.4 ± 0.6^a^	24.9 ± 2.6^a^	16.1 ± 0.8^b^
Total cholesterol (mg/dl) (serum)	56.1 ± 2.2^a^	54.6 ± 1.7^a^	97.4 ± 3.4^b^
Total cholesterol (mg/g tissue) (pancreatic islets)	1.16 ± 0.07^a^	1.17 ± 0.05^a^	1.65 ± 0.14^b^

Effect of short term feeding of high cholesterol peanut oil diet on fasting blood sugar, insulin, lipids and total cholesterol in pancreatic islets in Sprague Dawley rats (4 animals grouped for each diet type: total n = 12): Data are expressed as mean ± SEM. Mean values with different letters are significantly different at *P *< 0.05 level by Least Significant Difference test.
