# Supplementary material for: Restoring Mitochondrial Function: A Small Molecule-mediated Approach to Enhance Glucose Stimulated Insulin Secretion in Cholesterol Accumulated Pancreatic beta cells
Source: Sci Rep. 2016 Jun 10;6:27513. doi: 10.1038/srep27513 (PMC4901343; doi:10.1038/srep27513)
Supplement: Supplementary Information [file srep27513-s1.pdf]

Supplementary Information

Title:

Restoring Mitochondrial Function: A Small molecule Mediated Approach to Enhance Glucose Stimulated Insulin Secretion in Cholesterol Accumulated Pancreatic beta cells.

Authors:

Suman Asalla[1,2], Shravan Babu Girada[1], Ramya S.Kuna[1], Debabrata Chowdhury, Bhaskar Kandagatla[1], Srinivas Oruganti[1], Utpal Bhadra[3], Manika Pal Bhadra[4], Shasi Vardhan Kalivendi[4], Swetha Pavani Rao[4],Anupama Row[5], Ibrahim A[6], Partha Pratim Ghosh[7], Prasenjit Mitra[1*]

[1.] Dr. Reddy's Institute of Life Sciences, University of Hyderabad Campus, Gachibowli, Hyderabad, Telengana, India 500046

[2.] Dept. of Biochemistry, School of Life Sciences, University of Hyderabad, Gachibowli, Hyderabad, Telengana, India 500046.

[3.] Center of Cellular and Molecular Biology, Habsiguda, Uppal Road, Hyderabad, India, 500007

[4.] Indian Institute of Chemical Technology, Uppal Road, Tarnaka, Telengana, Hyderabad, India, 500007.

[5.] University of Hyderabad Health Center, University of Hyderabad, Gachibowli, Hyderabad, Telengana, India 500046.

[6]. Department of Biochemistry,' National Institute of Nutrition, Hyderabad 500007, India

[7] ·Microsoft India (R&D) Pvt.Ltd, Gachibowli, Hyderabad, Telengana, India 500032

Corresponding Author*:

Prasenjit Mitra.

Principal Scientist, Biology,

Dr.Reddy's Institute of Life Sciences,

University of Hyderabad Campus, Gachibowli, Hyderabad, Telengana 500046, India

Phone: 91-4066571500Fax: 91-4066571581, E mail: prasenjit.mitra01604@gmail.com; prasenjitm@drils.org

Supplementary Figure1a:

Total cholesterol in subcellular fraction comprising of mitochondria, endosome & secretory granules

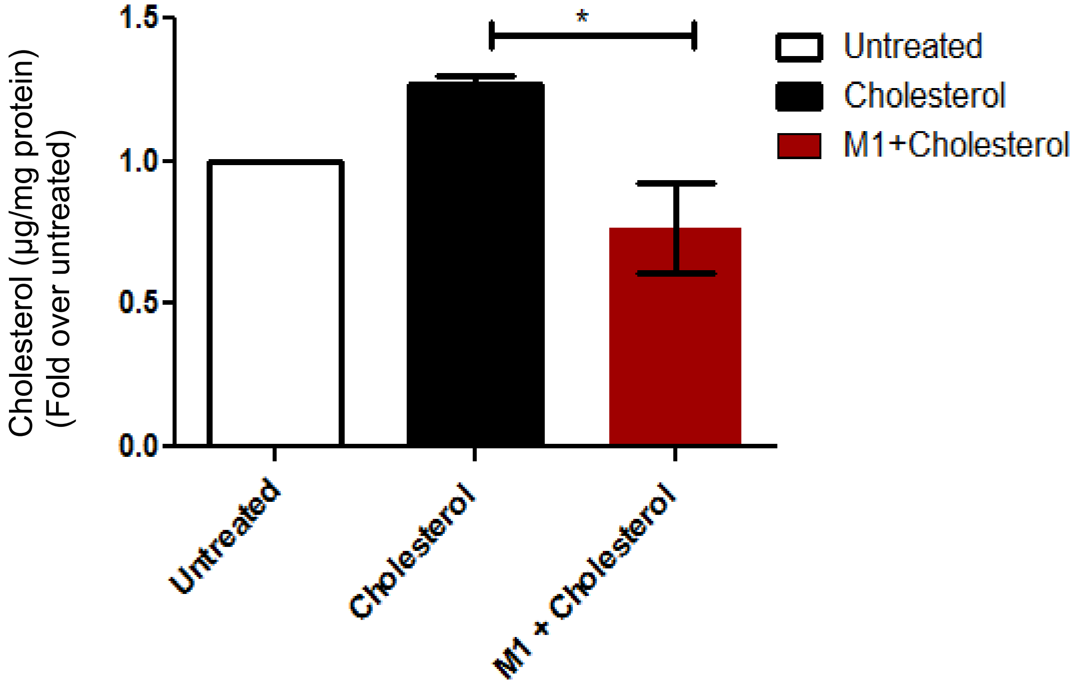

Supplementary Figure1a: Estimation of esterified cholesterol in subcellular fraction comprised of mitochondria, secretory granules and endosomes in untreated and cholesterol exposed pancreatic beta cells with or without the pre-treatment of small molecule M1. Sub-cellular fractionation was carried out following standard protocols and total cholesterol was extracted and measured through colorimetric assay. The normalized data measured as µg/mg protein were expressed as fold over untreated control and presented as mean ± SEM of 3 independent experiments.

Supplementary Fig 1b (i):

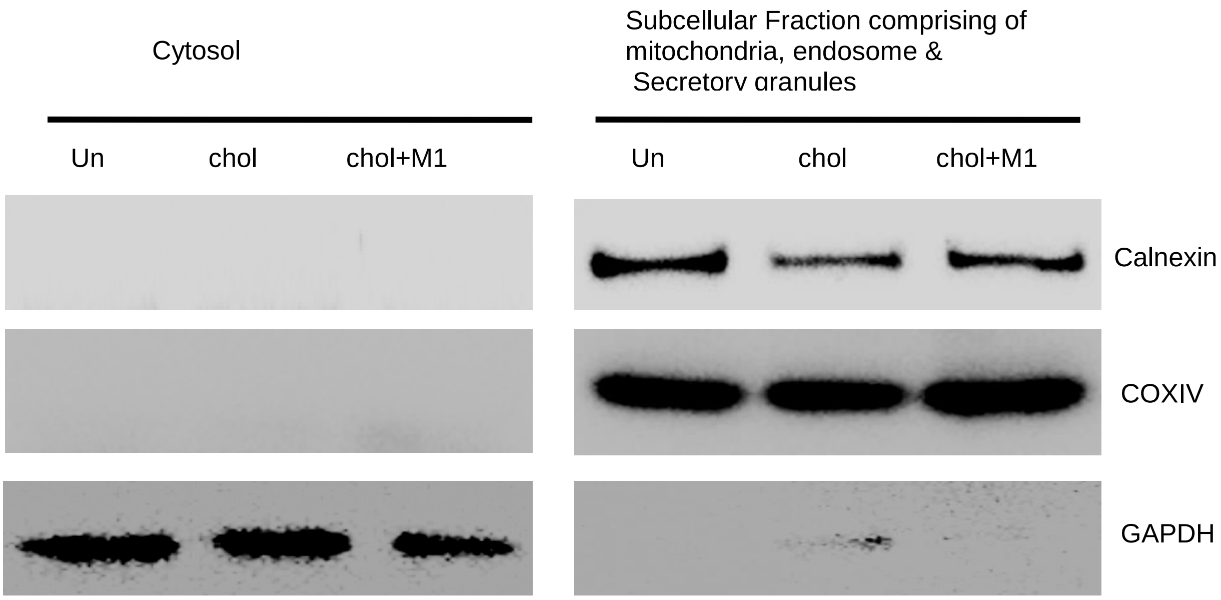

Supplementary Fig 1b (ii)

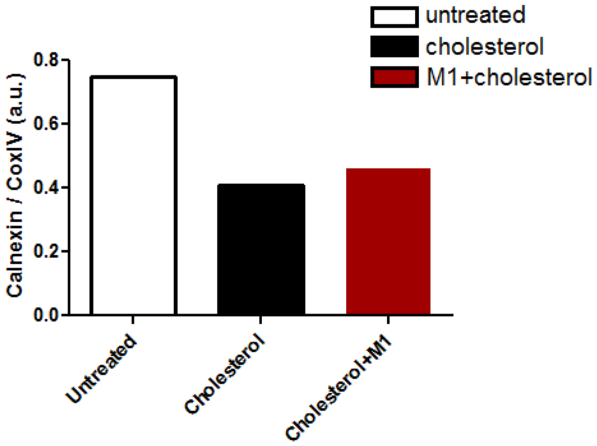

Supplementary Fig 1b: Western blotting of cytosolic and subcellular fraction comprised of mitochondria, secretory granules and endosomes isolated from untreated and cholesterol exposed pancreatic beta cells with or without the pre-treatment of small molecule M1. (i). Cytochrome oxidase IV (Cox IV) served as mitochondrial marker, Calnexin served as marker for rough ER and mitochondrial associated membrane (MAM), GAPDH serves as marker for cytosolic fractions. Densitometry showing the ratio of Calnexin: Cox IV (ii) is indicative of MAM presence in mitochondrial fractions of untreated and cholesterol enriched pancreatic beta cells with or without the pre- treatment with small molecule M1.

Supplementary Fig 1b (iii)

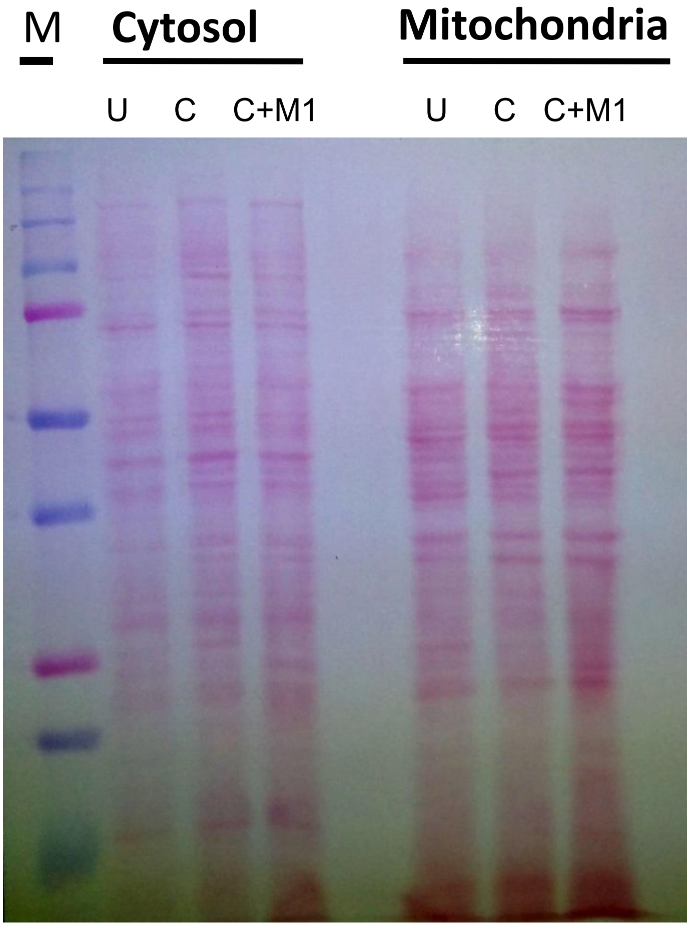

Supplementary Fig 1b(ii): Ponceau S staining depicting equal protein loading of untreated, cholesterol treated and cholesterol +M1 treated samples (cytosolic and mitochondrial fractions) that are subjected to western blotting with antibodies against marker proteins to ascertain the purity of the subcellular fractions.

Supplementary Figure 2:

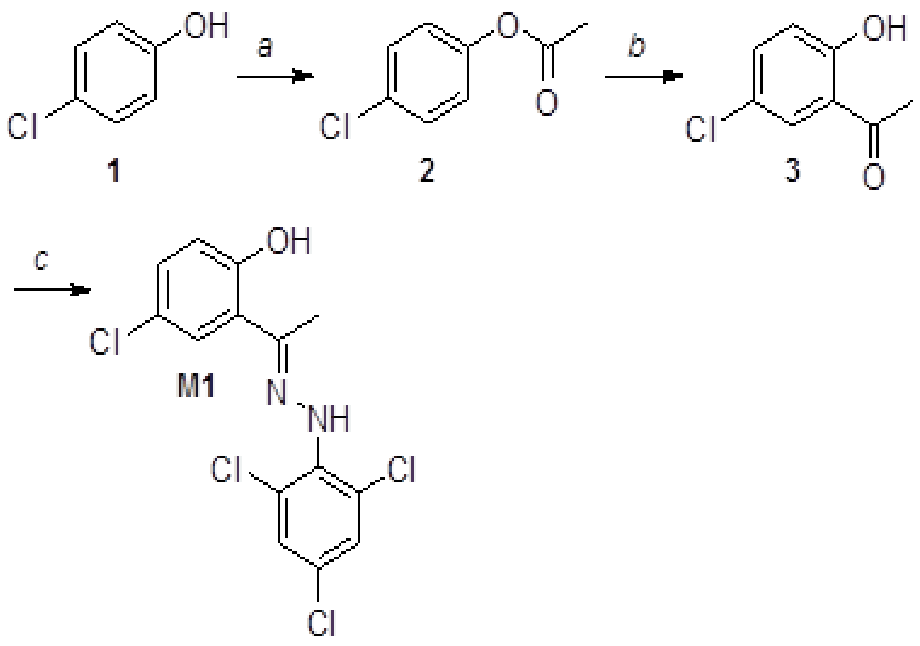

Supplementary Fig 2:Synthesis of 4-chloro-2-(1-(2-(2,4,6-trichlorophenyl)hydrazono)ethyl)phenol **(M1)** Reagents and conditions.(a) AcCl, $AlCl_3$, CH_2Cl_2, 0°C–rt., 4h; (b) Tf_2O, 80°C, 16h; (c) 1,3,5-Trichlorophenylhydrazine, AcOH, EtOH, 80°C, 1h. . 4-chlorophenyl acetate (2) obtained from acetylation of p-chlorophenol (1), was transformed to acetophenone (3) *via* Tf_2O mediated Fries rearrangement. Hydrazone formation between (3) and 2, 4, 6-(trichlorophenyl) hydrazine afforded convenient generation of M1

Supplementary Fig 3:

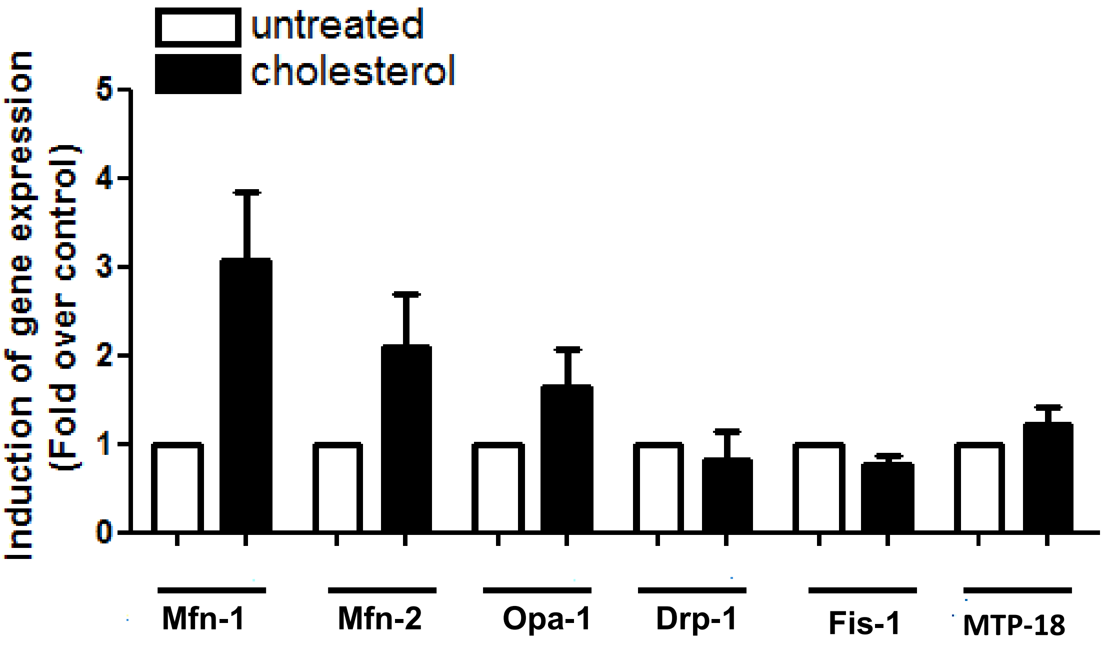

Supplementary Fig 3: Relative abundance of mitochondrial fusion and fission genes in cholesterol accumulated pancreatic beta cells as measured using $2^{-\Delta\Delta CT}$ method using GAPDH as invariant reference.

Supplementary Table 1

Patient code	FBS (mg/dL)	insulin(IU)	FH	Age(yrs)	TC(mg/dL)	TGL(mg/dL)	HDL(mg/dL)	LDL(mg/dL)
A1	114	18	0	48	133	75	50	68
B1	88	23	1	50	165	77	34	115
B2	78	23	1	39	209	98	41	148
A3	115	45	1	46	192	110	39	131
A4	104	28	0	40	209	88	43	145
A5	100	13	1	42	155	66	36	105
A6	108	43	1	52	216	65	34	169
B3	95	11	1	54	230	134	46	157
B4	92	16	1	41	168	118	42	102
A7	117	51	1	50	230	129	42	162
B5	135	34	0	49	235	147	46	159
A8	106	16	1	61	125	49	40	75
B6	148	90	1	52	209	120	34	151
B7	130	3	1	58	148	132	34	132
B8	310	54	1	48	192	87	35	139
A9	112	27	0	53	165	110	35	108
B9	237	64	1	55	240	290	38	144
B10	126	12.5	1	30	125	75	31	79
A10	101	22	0	50	150	88	34	98
B11	171	24	1	38	195	112	40	132
B12	128	60	0	43	225	132	39	153
A11	102	0	1	31	186	90	38	130
B13	285	54	1	57	182	98	32	130
B14	234	11	1	38	215	124	37	153
B15	137	11	0	46	254	280	46	152
A12	108	0	1	42	171	62	29	129
A13	104	0	1	50	270	170	47	189
B16	205	0	1	35	182	73	34	136
B17	128	6	0	58	187	82	34	136
B18	148	11	1	45	217	139	40	149
B19	348	24	1	55	272	154	43	198
A14	100	46	1	54	207	118	44	139
B20	73	3.3	0	20	214	160	36	146
B21	163	0	0	50	94	51	32	51
B22	186	25	1	47	180	216	35	101
B23	128	31	1	46	187	158	32	123
A15	104	33	0	49	180	76	32	132
B24	338	22	0	55	190	98	35	135
B25	182	19	0	58	159	76	34	125
B26	150	25	0	54	190	119	36	130

A16	117	3	0	50	240	116	43	173
B27	98	9	0	56	182	85	34	131
A17	102	4	1	41	162	115	37	102
A18	125	15	1	53	178	200	40	98
B28	144	0	0	55	226	298	39	127
B29	68	0	1	43	226	211	42	141
A19	117	22	1	39	190	120	33	133
B30	97	0	0	60	159	80	36	107
A20	115	9	1	57	226	155	39	156
A21	117	42	1	55	195	92	38	138
B31	212	0	1	52	215	132	34	154
B32	350	3	1	52	258	166	39	185
B33	172	24	1	55	172	88	32	122
B34	192	7	0	58	190	78	39	135
B35	172	38	1	57	192	101	33	138
B36	122	17	1	49	175	119	39	112
B37	170	21	1	40	190	128	41	123
A22	119	23	0	58	190	112	37	130
B38	153	11	1	47	185	116	35	126
B39	203	1	1	52	192	127	42	124
B40	75	1	1	54	221	140	43	150
A21	115	80	0	54	158	132	32	99
B41	90	7	0	49	175	121	40	110
B42	136	18	0	50	177	88	34	125
B43	220	0	0	37	190	132	43	120
B44	161	5	1	60	240	152	34	145
B45	126	4	0	45	180	119	42	167
A22	75	19	1	55	188	119	30	126
A23	139	18	0	59	158	79	39	125
A24	166	11	1	73	258	211	32	110
A25	112	8	0	54	188	162	33	122
A26	85	0	0	44	190	117	34	132
A27	170	36	0	49	208	89	40	150
A28	250	1	0	53	180	139	41	111
A29	77	0	0	51	188	110	39	127
A30	75	64	0	42	208	119	43	141
A31	156	0	1	56	190	101	38	131
A32	103	6	1	52	188	101	39	128
A33	385	38	1	50	176	89	39	119
A34	76	91	1	43	180	91	34	127
A35	85	26	0	58	170	68	35	121
A36	102	6	1	46	148	71	33	108
A37	75	18	0	23	180	88	39	123
A38	80	9	1	22	170	69	30	126
A39	91	0	1	28	231	77	40	175
A40	75	1	1	34	188	112	42	123

A41	88	11	1	45	210	169	43	133
A42	84	13	1	24	155	72	33	107
A43	95	22	1	38	305	197	48	217
A44	72	56	1	42	192	107	32	138
A45	82	13	0	35	207	110	32	153
A46	62	9	0	36	121	69	32	75
A47	65	0	1	22	162	66	31	117
A48	78	32	1	25	214	130	33	155
A49	76	8	0	22	206	170	36	136
A50	71	12.5	1	26	199	126	29	144
A51	72	8	0	24	143	118	38	81
A52	76	0	0	24	190	80	32	142
A53	103	91	1	36	188	92	37	132
A54	92	20	1	41	190	74	38	137
A55	92	70	1	58	154	96	30	104
A56	92	13	1	51	180	72	39	126
A57	88	10	1	37	170	92	34	117
A58	80	0	1	35	176	150	33	113
A59	106	15	0	46	188	395	43	66
A60	69	0	0	18	144	117	32	88
A61	66	0	0	24	195	92	29	147
A62	83	7	0	24	221	75	42	164
A63	76	8	0	24	192	144	31	132
A64	71	0	0	24	202	170	42	126
A65	81	8	0	33	180	117	37	119
A66	75	13	0	19	190	192	37	114
A67	75	1	0	18	173	109	35	116
A68	74	11	0	24	198	142	40	129
A69	70	0	0	45	267	126	48	193
A70	73	0	0	23	143	70	38	91
A71	125	19	0	44	224	110	39	163
A72	90	56	0	55	295	150	48	217
A73	71	8	0	18	136	52	25	100
A74	70	8	0	19	156	79	39	101
A75	67	21	0	20	129	59	31	86
A76	62	22	0	27	176	88	30	128
A77	80	8	0	23	202	102	32	149
A78	89	11	0	24	199	139	37	132
A79	88	5	0	26	170	88	32	120
A80	72	10	0	47	192	78	39	137
A81	95	13	0	52	172	65	39	120
A82	87	3	0	59	180	101	35	124
A83	79	3	0	61	210	128	39	145
A84	76	0	0	52	156	98	39	97
A85	82	15	0	45	219	128	41	150
A86	73	3	0	30	180	81	43	120

A87	75	5	0	50	201	118	40	137
A88	83	6	0	60	202	89	37	147
A89	84	21	0	45	170	102	32	117
A90	96	15	0	45	182	91	42	121
A91	74	5	0	50	165	75	31	119
A92	82	0	0	35	190	114	37	130
A93	80	0	0	49	215	108	39	117
A94	112	8	0	50	179	109	37	120
A95	70	30	0	60	220	88	40	162
A96	80	4	0	52	190	128	39	125
A97	105	0	0	42	205	60	43	150
A98	78	5	0	48	167	78	35	116

Supplementary Table 1: Description of Fasting Blood Sugar (FS), serum Lipid profile and family history of type 2 diabetes (FH) of volunteers assessed in this study. A volunteer whose parents suffer from type 2 diabetes was allotted a value of 1. A Volunteer with no family history of type 2 diabetes was allotted a value of 0.
